# Local production of eye drops in the hospital or pharmacy setting: considerations and safety tips

**Published:** 2023-05-22

**Authors:** Abeer H A Mohamed-Ahmed, Dan Kuguminkiriza

**Affiliations:** Research Fellow in Pharmacology and Clinical Trials Management: London School of Hygiene & Tropical Medicine, UK.; Pharmacist: Eye Drop Production Unit, Ruharo Eye Centre, Ruharu Mission Hospital, Mbarara, Uganda.

**Some eye drops can be locally prepared to improve patients' access to medication. Here is what you will need to consider.**

**Figure:**
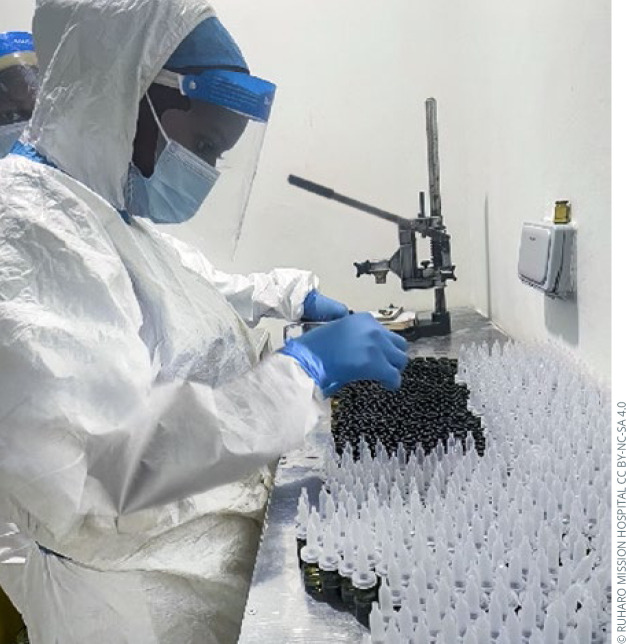
Eye drops preparation in Ruharo Mission Hospital. uganda

Most eye medicines for topical use are prepared by pharmaceutical companies using special automated mixing and filling machines. However, some eye drops can be prepared in a pharmacy setting in a hospital. There are several reasons^1^,^2^:

- Patients may be unable to afford commercially available eye drops, and locally prepared eye drops are often more affordable.
- Eye drops may be unavailable due to manufacturing shortages or because a product is discontinued; this can be overcome by preparing eye drops in hospital or pharmacy settings.
- Combinations of drugs that are not commercially available might be prepared locally; for example, eye drops combining anaesthetic and dilating agents.
- In some cases, the drug might be available in a formulation or strength that is not intended for ophthalmic use and needs to be adapted. One example is the preparation of amphotericin B eye drops from intravenous solution.
- Some eye drops need to be patient specific and therefore prepared individually; for example, autologous serum eye drops that are compounded using the patient's own serum.

The main ingredients for preparation of topical ophthalmic eye drops usually include:

- The active pharmaceutical ingredient (API), either as powder or as a concentrated solution.
- A solvent: either sterile water or buffer solution
- A preservative: an antimicrobial agent added to the eye drops to prevent microbial contamination of the liquid during use.

In general, eye drops are prepared in one of two ways:

1. Dissolving the active pharmaceutical ingredient/preservative (in powder form) in a suitable vehicle (either sterile water or a buffer solution).
2. Diluting a concentrated solution of the active pharmaceutical ingredient using sterile water or buffer solution.

There are several important elements that should be considered when preparing eye drops.

## 1. Sterilisation

Eye drops must be sterilised to ensure they are free of microbial contamination. The method of sterilisation depends on the stability of the drug at high temperature.

The options are:

- **Autoclaving.** Autoclaving is used to sterilise pharmaceutical products (solutions, suspensions, powder) which are stable at high temperature^1^. Eye drops in the final packaging (filled and sealed eye drop bottles) are usually sterilised at the end of the production process (terminal sterilisation) using autoclaving (saturated steam at 121–132°C) for 15 minutes to kill microorganisms.^1^
- **Filter sterilisation.** If the drug is not stable at high temperatures, eye drops in solution form can be sterilised by filtration through a 0.22 μm filter into a sterile final container. This method is called filter sterilisation and it should be conducted under aseptic conditions using a laminar flow cabinet. Filter sterilisation is not suitable for use with eye drops in suspension form, as the 0.22 μm filter will remove the finely dispersed drug particles and make the eye drops ineffective.

## 2. Inherent toxicity of the drug

The pharmacist should check the drug-specific data safety document (the drug safety data sheet) to get information about the toxicity of the drug. The pharmacist should adhere to the established guidelines for handling each drug.^3^

## 3. Removal of particulates

All compounded eye drop solutions should be filtered using a 5 μm filter to remove any visible particulate matter.^2^ This can be done using glass sintered filters or polypropylene fibre filters under minimal pressure. This pressure can be generated using either a hand-held or foot suction pump.

## 4. pH

The pH of eye drops is important for drug solubility and for the stability of some drugs.^3^ For optimal ocular comfort, it should be similar to the pH of natural tears (pH 7.4). However, sometimes it is not feasible to prepare eye drops with pH 7.4 due to drug stability or solubility issues. The acceptable pH range for eye drops is in the range of 6.5–7.8 to ensure patient comfort. A more acidic or alkaline pH can induce tearing, discomfort and pain.^4^,^5^ A suitable buffer can be used to control and maintain the pH of the eye drops during storage, such as citrate or acetate buffer.

## 5. Tonicity

Tonicity is defined as the ability of water to enter or exit through a membrane (e.g. cell membrane), via osmosis. The tonicity of the eye drops depends on the concentration of dissolved solutes (e.g. buffer salts and the active pharmaceurical ingredient). Ideally, the tonicity of eye drops should be similar to natural tears, which have a tonicity equal to 0.9% saline. In general, a range of 0.5–2% saline tonicity is well tolerated by most patients. Hypertonic solution (higher than the tonicity of 0.9% saline) can cause tearing. This increase in tear flow reduces the concentration of the drug in the eye, leading to reduction of drug efficacy.^3^,^6^ Hypotonic solutions (lower than tonicity of 0.9% saline) do not cause tearing, but might cause ocular discomfort.^3^,^6^

## 6. Preservatives

The addition of preservatives to multi-dose eye drops is crucial to prevent secondary contamination during storage and application.^7^ Several studies have reported severe ocular infections related to preservative-free ophthalmic preparations prepared in local pharmacy settings.^7^,^8^

Selection of suitable and safe preservatives is important.^7^ Eye drops that are intended for long-term use, e.g. for chronic eye conditions, should ideally be preservative free. These medications are not suitable for local production as they require highly specialised production facilities and special packaging, e.g., single-use packaging that avoids contamination during use. **Note:** some drugs, such as chlorhexidine, do not require the addition of preservative when prepared in the form of eye drops, because the drug itself acts as a preservative.

## 7. Stability

The drug should be stable in the selected solvent (e.g., buffer solution or sterile water). The stability of eye drops prepared within hospitals or pharmacies should be assessed according to the International Council for Harmonisation of Technical Requirements for Pharmaceuticals for Human Use (ICH) guidelines to determine the optimum storage conditions and drug shelf life.^9^ The shelf life (expiration date) should be determined based on the documented stability data and the potential for microbial contamination.^2^ The chemical stability of the active pharmaceutical ingredient(s), preservatives, other excipients (non-active pharmaceutical ingredients), and packaging should be considered when assessing the overall stability of the final ophthalmic product.^2^

## 8. Packaging and storage of the final product

The final container/packaging should be suitable for ophthalmic use and should not compromise the stability and efficacy of the topical preparation.^2^

Many compounded ophthalmic eye drops can be packaged in either sterile plastic bottles with integrated dropper tips (a standard eye drop container) or in glass bottles with separate droppers. The stability of some eye drops might be affected by the type of eye drops container used; for example, cyclosporine is absorbed by polyvinyl chloride, a polymer used in some plastic dropper bottles.

**Safety tips**

According to the American Society of Health-System Pharmacists (ASHP) Pharmacy-Prepared Ophthalmic Products guidelines, the following should be considered when preparing eye drops in the pharmacy/hospital setting^2^:

1. Adhere to aseptic techniques and sterilisation procedures to ensure that eye drops are sterile (free from microbial contamination).
2. Ask a colleague to double check your calculations of the amount of each ingredient that will be used in preparing the eye drops; this will minimise error.
3. All ingredients should be mixed in sterile, empty containers. When using more than one container for compounding a sterile preparation, each container should be labelled.
4. Compounding should be performed in a certified laminar airflow hood or, for a cytotoxic or hazardous product, inside a biological safety cabinet.
5. The compounded eye drops should be clearly labelled according to the hospital or pharmacy policy for prescription labelling. The label should contain information about the concentrations of active ingredients and preservatives and information about storage conditions, handling requirements, and expiration dates.
6. The storage instructions on the label should be clear. For example, room temperature means 15–25 °C, refrigerator means 2–8 °C, and freezer means below 0°C.
